# Corrigendum

**DOI:** 10.1111/acel.12823

**Published:** 2018-11-28

Song Z, Zhang J, Ju Z, Rudolph KL. Telomere dysfunctional environment induces loss of quiescence and inherent impairments of hematopoietic stem cell function. *Aging Cell*. 2012 Jun;11(3):449‐55. https://doi.org/10.1111/j.1474-9726.2012.00802.x.

In the article, “Telomere dysfunctional environment induces loss of quiescence and inherent impairments of hematopoietic stem cell function,” there are errors in Figure 3C,D and Figure 3E,F.

The representative FACS plots shown in Figure 3C were inadvertently taken from the experiment shown in Figure 2C. Representative examples of FACS plots (Fig. 3C) and a recalculated quantification of the cell cycle profiles (Fig. 3D) are shown below.

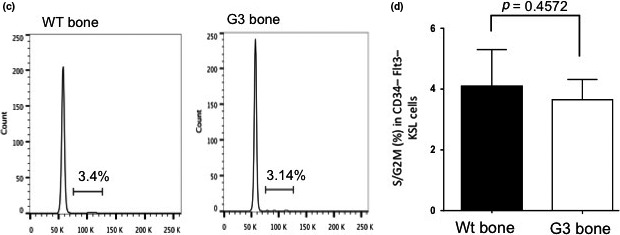

The figure legend should read: “(Fig. 3C,D) Representative FACS profile (C) and histogram showing the percentage of cells in S‐ and G2⁄M‐phase in wild‐type recipient‐derived LT‐HSC in the grafted bones of the indicated genotypes (D).” Figure panels “3C,D” should be referred to in the text instead of “3C.” The subsequent figure panels should be relabeled in the legend and in the text as “3E,F” instead of “3D,E.” The labeling of the *x*‐axis in Figuer 3E,F should read “WT stromal cells” and “G3 stromal cells” instead of “WT serum” and “G3 serum.” The FACS plots in Figure 2C show cell cycle profiles of hematopoietic stem and progenitor (KSL) cells. The p‐value in Fig. 1D for MPPs should state P=0.03 instead of P=0.0003.

The authors apologize for any confusion, which these errors may have caused.

